# Hospitals for Lady Probationers

**Published:** 1886-12-18

**Authors:** 


### HOSPITALS FOR LADY PROBATIONERS.

"A. R." writes:—"Will you kindly inform me of the best hospitals for lady probationers, terms, etc., where I could be trained for parish work?"

Ladies may now obtain the training necessary to qualify them for the profession of nursing, at most of the principal London and provincial hospitals, but the larger hospitals afford, perhaps, more general facilities for acquiring knowledge than the smaller ones, and the training may be had gratuitously by entering as an ordinary probationer. The St. John's House and Sisterhood, 6, Norfolk Street, Strand, receives ladies who are desirous of being trained for the nursing profession. This Sisterhood undertakes the nursing of King's College Hospital. The Westminster Training School and Home for Nurses, Queen Anne's Gate, S.W., does the nursing of Westminster Hospital. The regulations and system at St. Mary's Hospital were recently described in these columns. Another institution for training ladies is the Metropolitan and National Association for Providing Trained Nurses for the Sick Poor, 23, Bloomsbury Square, W.C. This Association only receives gentlewomen by birth and education, who are willing to undergo one year's hospital training. The matron of St. Thomas's Hospital would doubtless furnish "A. R." with the particulars of the Nightingale Nursing System. The London Hospital is another excellent institution, and Miss Luckës, the matron here, is a lady of wide experience and the authoress of a very useful manual for nurses. It would be well for any lady who contemplates entering a hospital to write to one of the Training Schools mentioned, or any other she may have reason to think well of, asking the Lady Superintendent to forward a copy of the Regulations for Probationers, and inquiring when any vacancy for a suitable applicant is likely to occur. This last is by no means an unimportant consideration for anyone desirous of being trained for a special purpose, to whom time may be an object. Candidates will find that the applications for vacancies are so numerous that the openings at the good Training Schools are nearly always filled up for months before they actually occur. By studying the Regulations obtained *carefully*, "A. R." will find that considerable variety exists at apparently similar institutions, both with regard to the opportunities for learning, the length of the hours on duty, and the arrangements made for the personal comfort of the Probationers.

